# Homing in on genomic instability as a therapeutic target in cancer

**DOI:** 10.1038/s41467-021-23965-5

**Published:** 2021-06-16

**Authors:** Craig M. Bielski, Barry S. Taylor

**Affiliations:** 1 Weill Cornell Medical College, New York, NY USA; 2 Department of Epidemiology and Biostatistics, Memorial Sloan Kettering Cancer Center, New York, NY USA; 3 Human Oncology and Pathogenesis Program, Memorial Sloan Kettering Cancer Center, New York, NY USA; 4 Marie-Josee and Henry R. Kravis Center for Molecular Oncology, Memorial Sloan Kettering Cancer Center, New York, NY USA; 5 Present Address: Loxo Oncology, a wholly-owned subsidiary of Eli Lilly, Inc., Stamford, CT USA

**Keywords:** DNA, Cancer genetics

## Abstract

While genomic instability is a hallmark of cancer, its genetic vulnerabilities remain poorly understood. Identifying strategies that exploit genomic instability to selectively target cancer cells is a central challenge in cancer biology with major implications for anti-cancer drug development.

The most common form of genomic instability in cancer is chromosomal instability (CIN), a continuous state of mitotic dysfunction that gives rise to karyotypic abnormalities and aneuploidy. Most human cancers exhibit some degree of CIN with consequences for tumor evolution and prognosis. CIN has been linked to other discrete sources of genomic instability such as whole genome doubling (WGD)^1^ and loss-of-function mutations in key tumor suppressor genes leading to dysregulated oncogenic signaling^2^. At the cellular level, CIN precipitates intratumoral heterogeneity and allows tumors to explore greater evolutionary space for fitter subclones that can subsequently expand under selective pressure^3^. CIN also causes the release of genomic DNA into the cytosol which triggers an immune response and activates inflammatory pathways that tumor cells can co-opt to promote metastatic dissemination^4^. These properties have significant clinical ramifications, as CIN is now recognized as a biomarker of poor prognosis across a diverse range of cancer types and both CIN and aneuploidy have also been implicated in multidrug resistance^5^, highlighting the importance of DNA copy number profiling in the clinical setting.

CIN is attributed in part to altered dynamics in the mitotic spindle that attaches to chromosomes and orchestrates the accurate separation of genetic material during mitosis. The spindle assembly checkpoint (SAC) safeguards against CIN by delaying the onset of anaphase until chromosomes are properly aligned and attached to the spindle. SAC defects can, therefore, lead to chromosome missegregation errors and aneuploid daughter cells. This phenomenon has been demonstrated in vitro. Treatment with small-molecule inhibitors of the SAC kinase MPS1 can induce CIN in near-diploid cell lines to generate aneuploid cell populations harboring random chromosomal aberrations^6^. Similar effects have been observed in transgenic mouse models with SAC deficiency, where overexpression of core SAC components such as *Bub1* or *Mad2* yields copy number gains and losses of whole chromosomes and enhanced tumor formation^7,8^. Given the relationship between SAC disruption and CIN, spindle proteins are attractive candidates for therapeutic development in CIN tumors. Nevertheless, therapies that target the underlying mechanisms that lead to CIN have proven elusive. While chemotherapies that target the mitotic spindle are commonly used to treat solid tumors, broad inhibition of spindle function as an anti-cancer therapy is complicated by the potential for toxicity^9,10^.

New evidence published recently in *Nature Communications* suggests that inhibition of a specific spindle protein may decrease cell viability in CIN tumor cells with little or no effect on diploid cells^11^. Reasoning that the altered spindle dynamics in CIN tumors may confer sensitivity to inhibition of SAC proteins, Marquis et al.^11^ systematically knocked down mitotic kinesin proteins in cancer cell lines, measuring the effects on cellular proliferation. The loss of one particular protein, KIF18A, was associated with significantly decreased viability in CIN tumor cells but not their stable, near-diploid counterparts. KIF18A plays a key role in spindle dynamics, regulating spindle microtubule growth and suppressing chromosome oscillations to facilitate the proper segregation of chromosomes. Despite this integral role in spindle maintenance, KIF18A appears to be dispensable in normal diploid cells, and notably transgenic *Kif18a* null mice are viable, albeit with developmental defects^12,13^. Taken together, these properties make KIF18A a potentially compelling therapeutic vulnerability specific to CIN tumors.

The antiproliferative effects of KIF18A loss in CIN tumor cells coincided with a number of mitotic errors, including prolonged mitotic delays, multipolar spindles, and centrosome fragmentation. While near-diploid tumor cells exhibited relatively minor mitotic delays upon KIF18A knockdown (KD), CIN tumor cells frequently experienced prolonged delays with large subpopulations of cells failing to complete mitosis altogether. CIN tumor cells dependent on KIF18A for cellular proliferation also showed a significant increase in multipolar spindle formation and centrosome fragmentation. These effects arose independently of mitotic delays, suggesting that KIF18A may function to maintain centrosome integrity and spindle bipolarity in the presence of CIN. Centrosome fragmentation in KIF18A KD CIN tumor cells did not require bipolar spindles, however, and was observed even in cells with monopolar spindles. Interestingly, chromosome fragmentation in these cells required dynamic microtubules, and it was therefore notable that each of the mitotic errors associated with KIF18A KD was attenuated by drugs that decreased microtubule dynamics and enhanced by drugs that increased microtubule dynamics. Moreover, these changes were not observed in chromosomally stable KIF18A KD cells, bolstering the potential of KIF18A as a therapeutic target specific to CIN tumors.

Two recent studies also identified the KIF18A vulnerability, using orthogonal approaches to identify tumors with ploidy abnormalities and their genetic dependencies based on complementary loss-of-function screens. Cohen-Sharir et al.^14^ leveraged publicly available genomic data from the Cancer Cell Line Encyclopedia (CCLE) to quantify aneuploidy in cancer cells based on copy number changes affecting whole chromosome arms. Core SAC genes *BUB1B* and *MAD2* ranked as the most preferential individual genetic dependencies in aneuploid cell lines, while the SAC as a whole scored among the most preferentially essential pathways. Yet, paradoxically, aneuploidy correlated with resistance to SAC inhibition in a trio of large-scale chemical screens. To better understand this phenomenon, the authors generated aneuploid cells by inducing cytokinesis failure in near-diploid cell lines and examined the effects of SAC inhibition on parental cells and their aneuploid derivatives. These engineered aneuploid cells exhibited altered dynamics and structural changes in the mitotic spindle which coincided with depleted expression levels of a single mitotic kinesin protein: KIF18A. Notably, *KIF18A* also scored among the top preferentially essential genes in aneuploid cells, and overexpression of *KIF18A* in engineered aneuploid cells induced sensitivity to SAC inhibition.

The second study from Quinton et al.^15^ examined the genetic dependencies conferred by whole genome doubling (WGD) in cancer cell lines. WGD is a common genomic aberration in human cancer that is associated with enhanced fitness and poor prognosis, yet relatively little is known about its concomitant genetic vulnerabilities. As with aneuploid cells, the two genes most strongly associated with essentiality in WGD-positive cell lines were *BUB1B* and *MAD2*, a result that underscores the established relationship between WGD and subsequent tolerance for CIN. *KIF18A* also scored as preferentially essential in WGD-positive cells, and these dependencies were validated in isogenic systems of matched diploid and tetraploid cell lines that differed only by their WGD status. Upon treatment with a SAC inhibitor, WGD-positive cells showed a marked increase in mitotic delays, chromosome missegregation errors, and micronuclei formation. Live cell imaging of these cells revealed similar effects following *KIF18A* depletion as well as changes in spindle morphology to accommodate the additional chromosomal burden following WGD. These effects rendered WGD-positive cells more susceptible to mitotic arrest in the absence of KIF18A, further supporting its promise as a therapeutic target in tumors with ploidy abnormalities.

Whether CIN and similar ploidy abnormalities constitute a novel therapeutic target in cancer remains to be seen. Genomic instability, and CIN in particular, is a nearly ubiquitous feature of human cancers, and a therapy that can exploit the fitness tradeoffs associated with CIN without disrupting the normal function of healthy diploid cells would represent a critical step forward in precision oncology. Collectively, these studies suggest that SAC protein KIF18A may be a therapeutic target that is specifically required in cells characterized by CIN or other related ploidy abnormalities (Fig. 1). It is worth noting, however, that these studies differ markedly in terms of how they define the genomic instability that leads to KIF18A dependency. Indeed, there is no consensus definition of CIN as it relates to the tumor genome. It is unlikely a single biologically homogeneous genotype, but rather many variations with distinct biological states and context-specific phenotypes. And while these studies are certainly exciting, they are just the first step toward decoding the unique genetic dependencies associated with CIN. The therapeutic index of KIF18A inhibition in cancer patients with CIN tumors is still unknown, especially given its role in maintaining essential mitotic machinery and other potential phenotypic defects^13^. Nevertheless, these studies provide a valuable blueprint for target discovery leveraging computational analysis of increasingly complex genotypes and represent an important advance in our understanding of CIN in cancer.

**Fig. 1:**
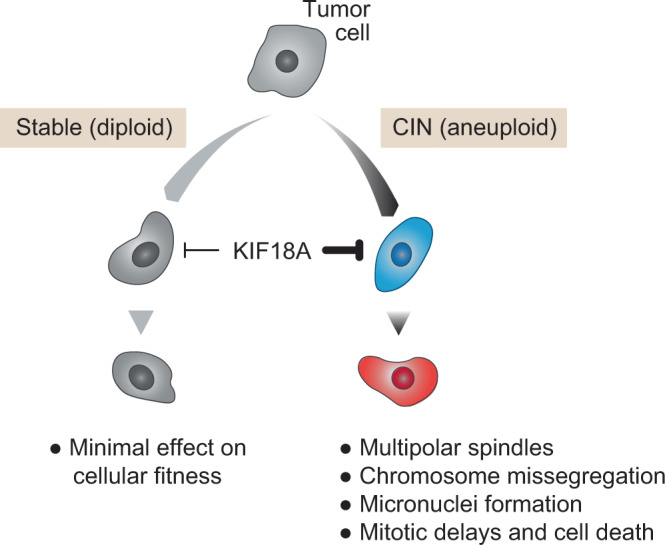
KIF18A dependency in CIN tumor cells. CIN tumor cells (via one of several forms of ploidy defects) are uniquely dependent on KIF18A, the inhibition of which leads to myriad phenotypic abnormalities absent in diploid cancers.
